# Experimental study on mechanical response of soft soil freezing in underground excavation using artificial ground freezing method

**DOI:** 10.1371/journal.pone.0350241

**Published:** 2026-06-22

**Authors:** Lu Han, Lingbing Yi, Peng Zhang, Lujie Chai, Ziming Cheng

**Affiliations:** 1 School of Transportation Engineering, Huanghe Jiaotong University, Jiaozuo, China; 2 China Transportation Construction Co., Ltd., Beijing, China; 3 Tsinghua University, Beijing, China; 4 China Railway Tunnel Bureau Group (Shanghai) Special High-Tech Company Limited, Shanghai, China; 5 Hainan University, Haikou, China; 6 North China University of Water Resources and Electric Power, Zhengzhou, China; Henan Polytechnic University, CHINA

## Abstract

To ensure the stability of the artificial frozen wall during the underground excavation of a proposed subway station using the artificial ground freezing method in soft soil strata, and to guarantee the construction safety of the proposed station under-crossing beneath an existing operating station. Based on the project of a proposed subway station under-crossing an existing station via underground excavation with artificial ground freezing method, four typical soft soils, namely silty clay, mucky soil, residual cohesive soil, and fully weathered ignimbrite, were selected to conduct systematic physical and mechanical tests of frozen soil under the temperature range of −20°C to −5°C. The influence mechanism of temperature and confining pressure on the thermodynamic behavior of frozen soil was revealed through the transient hot wire method, unidirectional frost heave and thaw settlement test, and triaxial shear test. The results show that the thermal conductivity of soil samples increases significantly at low temperatures, with the largest increase observed in fully weathered ignimbrite and the smallest in mucky soil. The freezing temperature of soil samples under natural moisture content ranges from −2.25°C to −0.8°C, with the lowest value recorded for residual cohesive soil. The creep of frozen soil exhibits obvious stress dependence: it presents typical three-stage creep characteristics at the stress level of 0.5σs, and its long-term strength is approximately 0.5–0.7 times the instantaneous strength. In engineering practice, lower freezing temperature or strict control of loading duration should be adopted for soil layers with high creep potential. The triaxial shear strength of frozen soil increases with the decrease of temperature, and the most significant increase occurs in the temperature range of −5°C to −10°C. The strength of the tested soils ranks as follows: fully weathered ignimbrite > residual cohesive soil > silty clay > mucky soil. The influence of confining pressure on strength varies with soil types. During construction, differentiated freezing and support control measures shall be implemented according to the characteristics of soil layers, and monitoring shall be strengthened for sections with abnormal confining pressure response. The research results can provide an important basis for design optimization and construction risk control of subway underground excavation projects using artificial ground freezing method in soft soil areas.

## 1. Introduction

With the acceleration of global urbanization, the development of urban underground space has become a core strategy for alleviating traffic congestion, improving land use efficiency, and promoting the sustainable development of cities [1–3]. Underground engineering construction in water-rich soft strata has long been faced with challenges such as stratum instability, water and sand inrush, and excessive surface deformation. The Artificial Ground Freezing (AGF) method, with its excellent water-sealing performance, strong stratum adaptability, and environmental friendliness, has become the core construction method for underground engineering in water-rich strata [3–6]. In recent years, scholars at home and abroad have carried out extensive research on key issues including the mechanical properties of frozen soil, evolution of freezing temperature field, construction deformation control, and engineering disaster prevention and control, which has laid a solid theoretical and experimental foundation for the engineering application of this technology.

As a four-phase composite material, the mechanical behavior of frozen soil is controlled by the coupling of multiple factors such as mesoscopic structure, temperature, and stress state [4,7,8]. Zhou et al. [7] combined plane strain tests with discrete element simulations to reveal the regulation law of particle rotation moment and mesoscopic friction parameters on the macroscopic mechanical behavior of frozen sand. Lai et al. [3] confirmed through directional shear tests that the soil-ice composite formed by pressurized freezing has essentially different mechanical properties from conventional unpressurized frozen samples, providing mechanical parameters for the low-carbon design of freezing projects. Aiming at the mechanical properties of different types of frozen soil, Qu et al. [9] established a constitutive model suitable for frozen peat soil; Lijith et al. [10] developed a modified temperature-controlled direct shear apparatus and identified the key influencing factors of the shear strength of frozen fine sand; Sun et al. [11] quantified the deterioration effect of freeze-thaw cycles on the structural strength of expansive soil in seasonally frozen regions; Zhao et al. [12] constructed a high-precision prediction model for the unconfined compressive strength of salinized frozen soil based on machine learning; Li et al. [13] proposed an in-situ evaluation method for artificial frozen soil strength based on electrical resistivity, revealed the strong linear correlation between unconfined compressive strength, deformation modulus of frozen soil and resistivity, and clarified the coupled influence of water-ice phase transition and pore structure evolution on the mechanical and electrical properties of frozen soil through BET and SEM tests, which provided a new non-destructive way for accurate strength evaluation of frozen soil in engineering. In terms of the construction of frozen soil constitutive models, Chang et al. [14] established a mesomechanics-based constitutive model for frozen coarse sand; Zhang et al. [15] derived a creep model of ice considering freezing pressure and confining pressure; Sun et al. [16] revealed the influence of confining pressure on the strength and deformation law of pressure-frozen ice through low-temperature triaxial tests, providing constitutive support for the numerical simulation of frozen soil engineering.

The shear characteristics of the frozen soil-concrete interface are core parameters for the structural design of cold region engineering and freezing engineering [17,18]. Xie et al. [17] quantitatively analyzed the regulatory effect of interface roughness on the freezing strength of the frozen soil-concrete interface for the first time, and clarified the law that the peak interface strength increases significantly with the increase of roughness. Zhang et al. [18] combined digital image correlation technology to reveal the evolution law of interface shear behavior under the coupling effect of multiple factors, and elucidated the differential deformation characteristics of interface strain hardening and softening at different temperatures, providing key experimental support for the interface design of underground structures.

Accurate prediction of the freezing temperature field is the core premise for the design of freezing engineering, and its formation process is essentially a hydro-thermo-mechanical multi-field coupling process of the stratum [2,19,20]. In terms of analytical calculation, Hu et al. [19] derived a generalized analytical solution for the steady-state temperature field of double-circle-piped freezing based on conformal mapping, which improved the analytical theoretical system of artificial freezing temperature field. Aiming at the multi-field coupling problem of complex strata, Gao et al. [2] and Zhu et al. [21] established hydro-salt-thermo-mechanical multi-field coupling theoretical models for sand-clay composite strata and coastal sandy strata under seepage conditions respectively, and quantified the weakening effect of seepage on freezing effect. Sun et al. [5] revealed the influence law of seepage velocity on the evolution of the temperature field of the frozen wall through laboratory model tests, and proposed a method for determining the closure time of the frozen wall and the critical seepage velocity. Yan et al. [20] established a systematic evaluation method for freezing engineering effect by combining field monitoring, analytical calculation and numerical simulation, and verified its reliability through engineering practice.

The optimization of the combined cement improvement and artificial ground freezing method, as well as the prevention and control of frost heave and thaw settlement, are key research directions for the application of freezing engineering [1,22,23]. Zhang et al. [1] clarified that 10% is the optimal cement content, which can maximize the thickness of the frozen wall and promote the uniform distribution of the temperature field, and also revealed the influence law of excavation rate on stratum settlement. Hua et al. [22] and Wang et al. [24] respectively clarified the regulatory law of cement content and curing age on the frost heave and thaw settlement characteristics and strength evolution of improved soil through laboratory tests, and confirmed that 8% cement content has the optimal inhibition effect on frost heave and thaw settlement. In terms of frost heave deformation calculation, Wang et al. [6] derived a calculation formula for ground deformation caused by artificial frozen soil frost heave based on stochastic medium theory, and defined the influence range of freezing deformation. Wang et al. [23] optimized the design parameters of the frozen wall through numerical simulation, and confirmed that 12% cement content combined with brine temperature of −31°C can reduce surface frost heave by 73%. Aiming at the deformation control of adjacent construction in water-rich strata, Li et al. [25] systematically analyzed the influence law of key construction parameters on the settlement of existing structures based on a metro close under-crossing project, optimized the construction parameters and verified their effectiveness through field monitoring.

In terms of the calculation of mechanical parameters for freezing engineering and the research on creep characteristics of frozen soil, Han et al. [26] obtained the measured data of frost heave force on segments during the whole freezing construction process through in-situ monitoring, and revealed the core influencing factors of frost heave force evolution. Liu et al. [27] established a calculation formula for tunnel frost heave force considering the synchronous damage of tunnel lining and surrounding rock, realizing the accurate calculation of frost heave force. Yao et al. [28] carried out uniaxial creep tests of artificial frozen soft soil under different temperatures and stress levels, improved the traditional ant colony algorithm by optimizing the pheromone fuzzification coefficient, and established a fuzzy random evaluation system for the creep model of frozen soft soil, providing a technical method for the optimal selection of frozen soil creep models in metro tunnel freezing construction. Chen et al. [29] systematically studied the creep behavior of deep frozen clay under multiple temperature and stress levels through laboratory tests, revealed the differentiation law of attenuated and non-attenuated creep of frozen soil, constructed a creep analytical model of frozen wall based on Burgers rheological model, and verified the reliability of the theoretical model through field measured data, which provided an effective reference for creep deformation control and construction safety guarantee of frozen wall.

The evolution mechanism of water and sand inrush and soil erosion disasters in underground engineering of water-rich sand strata is the core basis for disaster prevention and control [30–32]. Peng et al. [31] revealed the core controlling factors and evolution mechanism of sand erosion caused by tunnel leakage in water-rich sand strata through laboratory model tests. Jiang et al. [32] identified three types of hazard-inducing structures for water and sand inrush in sandy dolomite strata, and revealed the disaster evolution mechanism and key influencing factors under different working conditions. Zhang et al. [30] took a water and sand inrush accident in Shanghai Metro as a case study, clarified the core cause of the accident and the effectiveness of treatment measures, providing practical experience for the risk prevention and control of similar disasters.

The experimental research on the mechanical properties of rock and soil under complex stress states and the development of equipment are important research directions in the field of frozen soil mechanics [33–35]. Wang et al. [33] developed a true triaxial apparatus with three-dimensional rigid boundaries, which solved the mutual interference problem of traditional rigid plate loading, and provided equipment support for the mechanical property test of rock and soil under true three-dimensional stress state. Aiming at the mechanical behavior of soil under true triaxial stress state, Silvani et al. [36] revealed the influence law of intermediate principal stress on the strength of industrial by-product improved soil; Li et al. [37] analyzed the effect of strain localization on the strength and deformation characteristics of aeolian sand; Dong et al. [38] clarified the damage evolution law and failure mode transformation characteristics of sandstone under true triaxial cyclic loading. For the strength characteristics of frozen soil under complex stress, Yao et al. [35] proposed a strength criterion for artificially frozen sand considering temperature effect; Zhang et al. [39] established a strength criterion for frozen silty clay considering the effect of intermediate principal stress; Huang et al. [34] constructed a constitutive model of frozen sand under true triaxial stress state, which improved the strength and deformation calculation theory of frozen soil under complex stress.

In terms of engineering application and comprehensive evaluation of the artificial ground freezing method, Park et al. [8] confirmed that frozen soil can effectively inhibit the deformation of the retaining structure through a deep foundation pit freezing engineering case, deepening the engineering application cognition of freezing technology in complex sedimentary strata. Bublik et al. [40] revealed the mechanism of salinity on the thermophysical properties of soil and the formation process of frozen wall, providing a theoretical basis for the design of freezing engineering in salinized soil strata. Zhou et al. [3] systematically sorted out the application characteristics and technological innovation achievements of the artificial ground freezing method in the past 20 years, discussed the technology development trend and future core research directions, and provided a review reference for the subsequent development of this technology.

In summary, existing studies have constructed the theoretical and technical system of the artificial ground freezing method from the dimensions of frozen soil mesomechanics, macroscopic constitutive model, multi-field coupling theory, engineering deformation control, and disaster prevention and control. However, there are still obvious research gaps: Firstly, most existing studies on the mechanics of frozen sand are based on conventional unpressurized frozen samples, and there is still a lack of systematic experimental research on the mechanical behavior of pressurized frozen sand under complex stress paths, especially the strength evolution law under directional shear [4,35]. Secondly, for the combined cement improvement and artificial ground freezing method, there is still a lack of systematic theoretical analysis and engineering verification on the regulation mechanism of cement improvement on the characteristics of frozen wall and the coupling influence of excavation rate on the foundation settlement of upper buildings [1,23]. Thirdly, the accurate calculation theory of frost heave and thaw settlement deformation under complex seepage strata and multi-circle pipe freezing conditions still needs to be further improved [2,6].In addition, blind deconvolution and sparse representation methods have also been introduced into fault diagnosis, such as the blind deconvolution algorithm based on a multi-node network structure [41] and the adaptive resonant band detection method utilizing cyclostationarity and the sparse constraint of the L2/L1 norm [42].

Aiming at the above research gaps, this paper takes the artificial freezing engineering in water-rich sand strata as the research background, adopts a combination of laboratory tests, theoretical analysis and numerical simulation to systematically study the mechanical behavior of pressurized frozen saturated sand under directional shear, the regulation mechanism of cement improvement on the characteristics of frozen wall, and the influence law of freezing tunnel excavation on the foundation settlement of upper buildings. The corresponding strength criterion and deformation calculation method are established, and the optimization method of key design parameters is clarified. The research results can improve the mechanical theoretical system of frozen sand under complex stress conditions, and provide theoretical support and practical guidance for the design optimization and safety control of artificial freezing engineering in urban water-rich strata.

## 2. Overview of project

**Fig 1:**
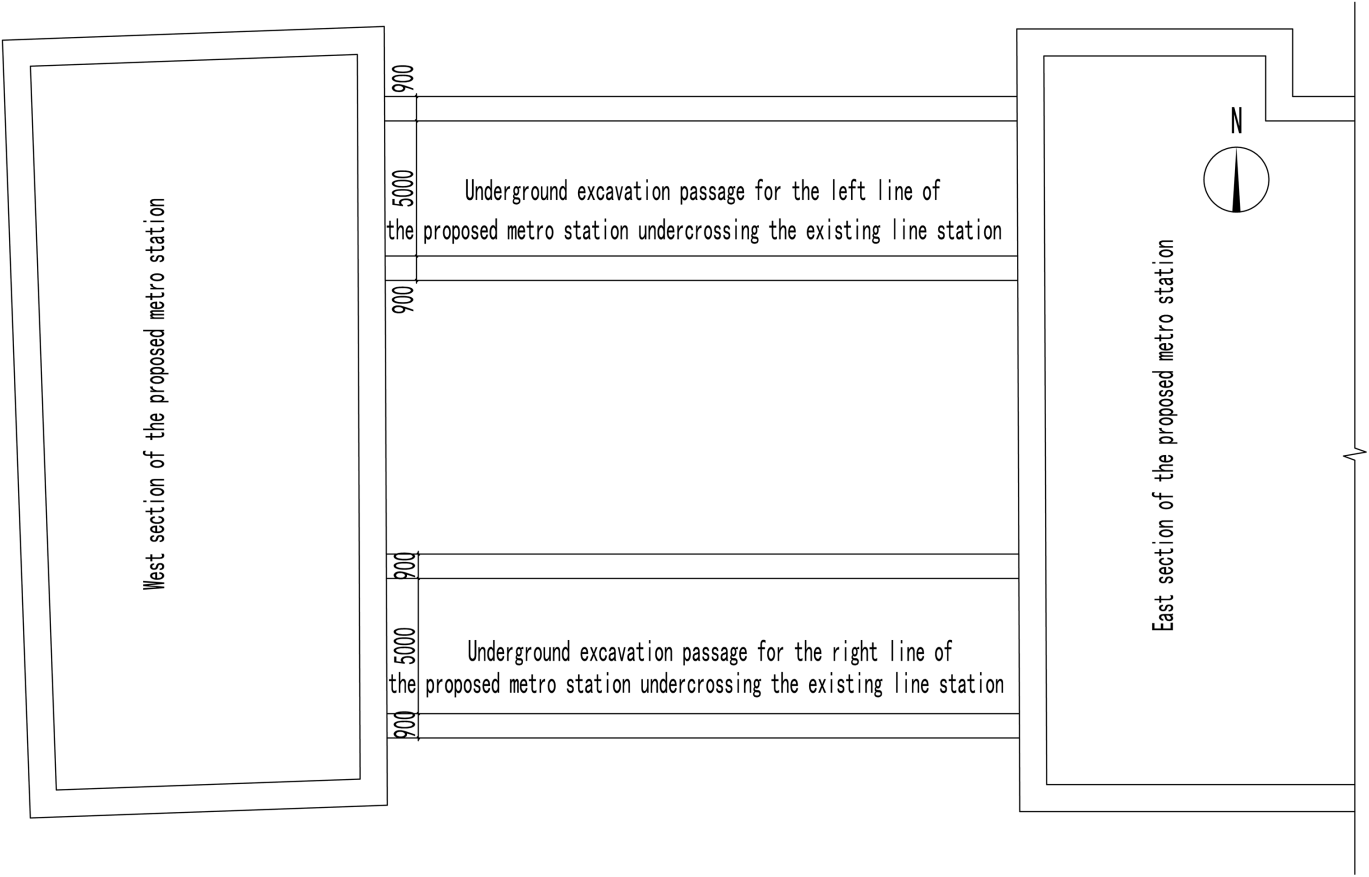
Planar location diagram (Unit: mm).

**Fig 2:**
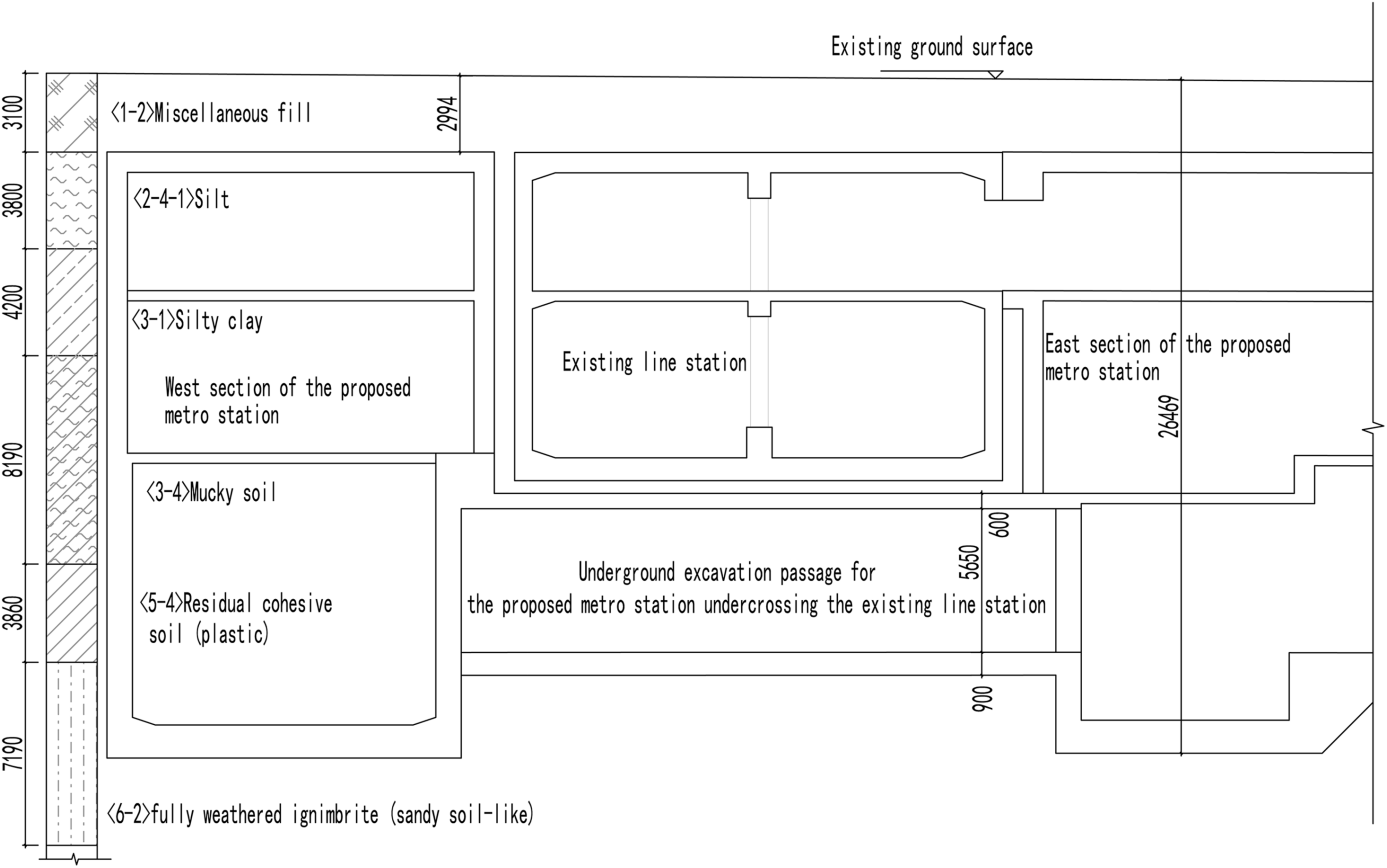
Sectional location diagram (Unit: mm).

The proposed subway station, located at a crossroad, is constructed by underground excavation with artificial ground freezing method to under-cross an existing operating metro station. The underground excavation tunnel adopts a rectangular section, with a net inner size of 5.65m (height) × 5.00m (width) and an excavation size of 8.08m × 7.60m. The distance between the structural roof of the under-crossing section and the bottom cushion of the existing station is about 0.3m (excluding the thickness of the cushion). The strata within the influence range from top to bottom are: silty clay, mucky soil, residual cohesive soil (plastic), and fully weathered ignimbrite (sandy soil-like). The plan and section diagrams are shown in Figs 1 and 2 respectively.

## 3. Test design

### 3.1 Sampling

The test samples are Φ50 × 100 mm, processed from undisturbed rock cores (length error ≯±1 mm, diameter error ≯±0.5 mm). After weighing and numbering, the samples were cured at negative temperature for 48 hours in accordance with the specifications, and then the following indexes were measured: thermal conductivity, freezing temperature, uniaxial compressive strength (including elastic modulus, stress-strain relationship), uniaxial creep, and triaxial shear strength. The sampling for frozen soil tests is shown in Table 1.

**Table 1:** Sampling list for frozen soil tests.

stratigraphic subdivision	Soil layer	Natural unit weight/kN·m ⁻ ³	Compression modulus/MPa	Cohesion/kPa	Internal friction angle/°	Poisson’s ratio
**3−1**	**silty clay**	19.90	5.90	23.80	14.60	0.32
**3-4**	**mucky soil**	18.20	4.80	10.20	5.80	0.38
**5−4**	**residual cohesive soil (plastic)**	18.90	8.70	26.80	15.70	0.33
**6−2**	**fully weathered ignimbrite (sandy soil-like)**	18.90	12.30	24.70	25.80	0.31

### 3.2 Thermal conductivity test

The transient hot wire method was selected for the thermal conductivity test, because it is suitable for heterogeneous materials and can quickly capture the transient thermal response characteristics of frozen soil. Compared with the steady-state method, the transient method can avoid the test error caused by water migration in frozen soil.

**Fig 3:**
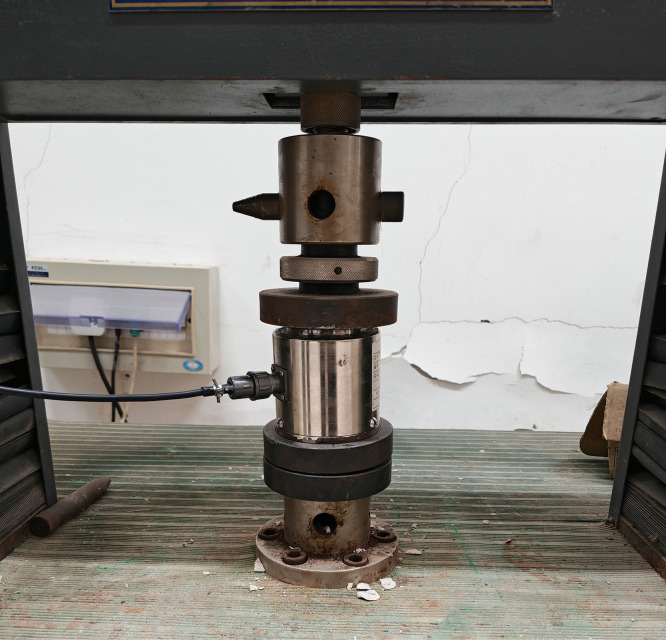
Specimen pressing.

The thermal conductivity was measured by a TC3000E thermal conductivity analyzer with transient hot wire method, with an instrument range of 0.005 ~ 10 W/(m·K), a temperature range of −60 ~ 120°C, and an accuracy of ±3%. The samples were prepared by static pressure pressing method, and the probe was placed between two identical samples for testing. The thermal conductivity of the four soil samples with natural moisture content was tested under normal temperature (20°C for thawed soil) and low temperature (−10°C for frozen soil). The sample preparation is shown in Fig 3.

### 3.3 Freezing temperature test

**Fig 4:**
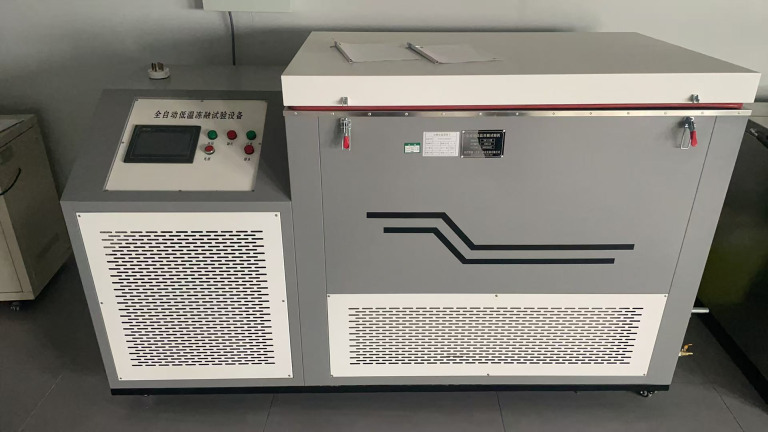
Frost heave and thaw settlement test equipment.

**Fig 5:**
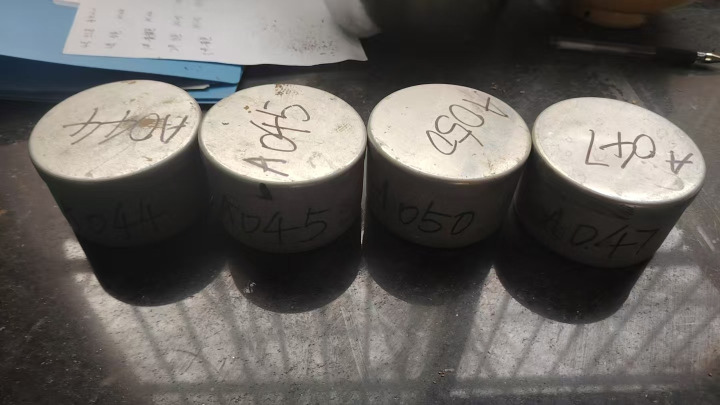
Frost heave and thaw settlement test procedure.

The test was carried out in accordance with Test on Physical and Mechanical Properties of Artificial Frozen Soil in the Geotechnical Test Standard of Coal Industry of the People’s Republic of China (MT/T 593.2−2011), to determine the freezing temperature of the four soil samples under natural moisture content at −10°C. The samples were prepared by the sample compaction method: the dried and crushed soil samples were prepared according to the set moisture content, with a diameter of 100 mm and a height of 120 mm, filled in five layers with 30 blows for each layer. After preparation, the samples were loaded into a prefabricated mold with an outer diameter of 99 mm, an inner diameter of 80 mm and a height of 160 mm, and the unidirectional frost heave and thaw settlement test in a closed system was carried out within the temperature range of −40°C to 60°C. The main equipment includes TMS9018 constant temperature chamber, CR3000 data acquisition instrument, DA-15 high-precision displacement sensor, freeze-thaw device barrel, TMS8037 cold bath and computer. Test procedure: constant temperature at 1°C in the environmental chamber for 6h → adjust to freezing temperature for more than 12h → adjust to 5°C for natural thawing → stop the test and save the data after the reading is stable for 1h. The test process is shown in Figs 4 and 5.

### 3.4 Uniaxial compressive strength test of frozen soil

The uniaxial strength and creep tests of frozen soil were carried out on a self-developed WDT-100 frozen soil testing machine at different temperatures (−5°C, −10°C, −15°C) to obtain the strength, elastic modulus and stress-strain curve. The uniaxial creep test was conducted in accordance with Test on Physical and Mechanical Properties of Artificial Frozen Soil (MT/T 593.6–2011), with three levels of load: σ = 0.3σs, 0.5σs, 0.7σs (σs is the instantaneous compressive strength of frozen soil), and the multi-sample method was adopted.

### 3.5 Triaxial shear test of frozen soil

The triaxial shear test of frozen soil under natural moisture content with different temperatures, confining pressures and loading rates was carried out by using MTS fatigue testing machine and special constant temperature environmental chamber. All samples were remolded soil, with moisture content set according to liquid limit, plastic limit and optimal moisture content, constant dry density, and size of Φ50mm×100 mm.

**Fig 6:**
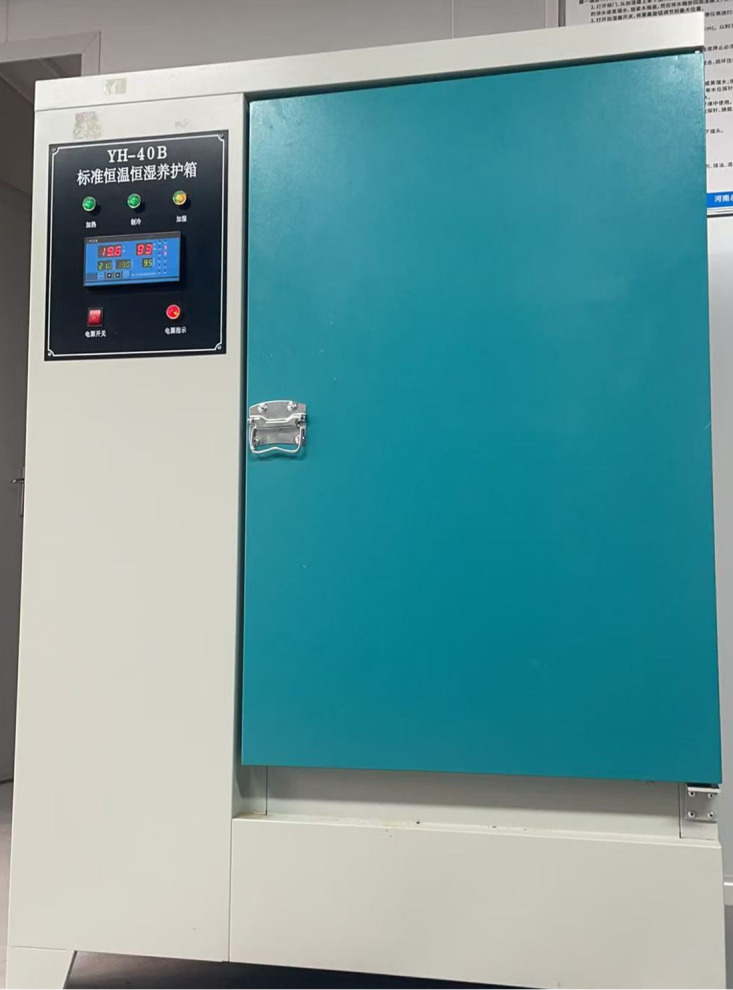
Special constant temperature environmental chamber for specimens.

**Fig 7:**
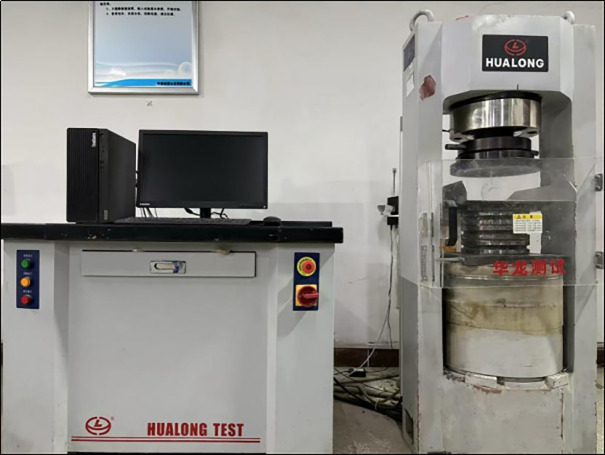
MTS fatigue testing machine.

To prevent water migration, the prepared samples were quickly frozen in an environmental chamber at −30°C for 3h, and then cured at the test temperature for 24h. The MTS program was set to control loading/unloading. The shear was controlled by strain rate, and the test was terminated when the axial strain reached 20% or the sample was damaged. The test process is shown in Figs 6 and 7.

## 4. Result analysis

### 4.1 Thermal conductivity

**Fig 8:**
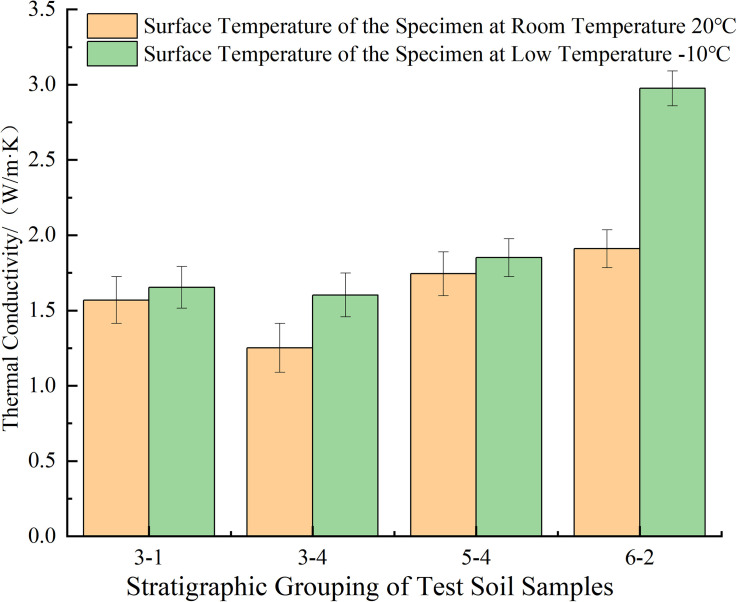
Thermal conductivity test results.

The test results of thermal conductivity of soil samples are shown in Fig 8. The thermal conductivity of soil samples tends to increase with the decrease of freezing temperature. Among them, the fully weathered ignimbrite has the largest increase in thermal conductivity, which is derived from the difference in mineral composition and pore structure: the fully weathered ignimbrite has low porosity and diverse minerals, which promotes the dense formation of ice crystals and enhances heat conduction; in contrast, the high porosity of silty clay leads to the dispersion of ice crystals, resulting in a smaller increase in thermal conductivity. This is consistent with previous research results.

### 4.2 Freezing temperature

**Fig 9:**
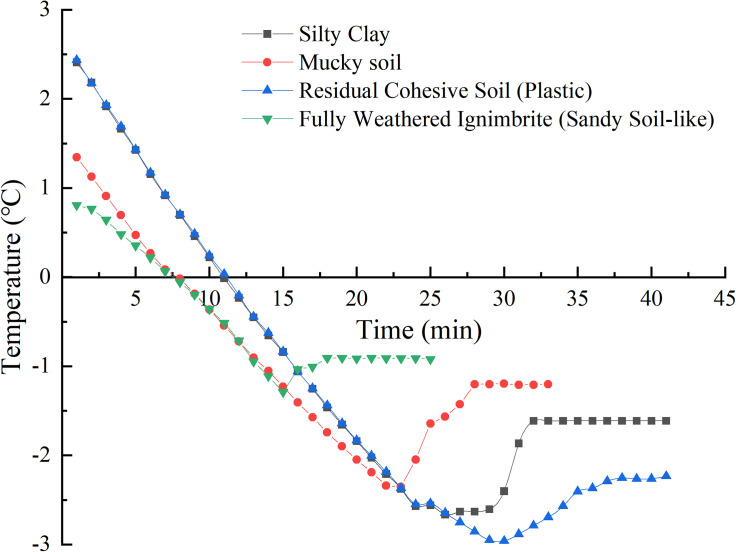
Freezing temperature of each soil sample under natural moisture content at −10°C.

The time-dependent temperature curves of the four soil samples under natural moisture content at −10°C are shown in Fig 9. It can be analyzed from the figure that the freezing temperatures of the four soils are as follows: (a) the freezing temperature of silty clay is −1.7°C, (b) the freezing temperature of mucky soil is −1.25°C, (c) the freezing temperature of residual cohesive soil (plastic) is −2.25°C, and (d) the freezing temperature of fully weathered ignimbrite (sandy soil-like) is −0.8°C. The residual cohesive soil has the lowest freezing temperature. The analysis shows that the clay particles and organic matter in the residual cohesive soil adsorb water and reduce the freezing point, while the high moisture content of mucky soil raises the freezing temperature.

### 4.3 Uniaxial compressive strength of frozen soil

**Fig 10:**
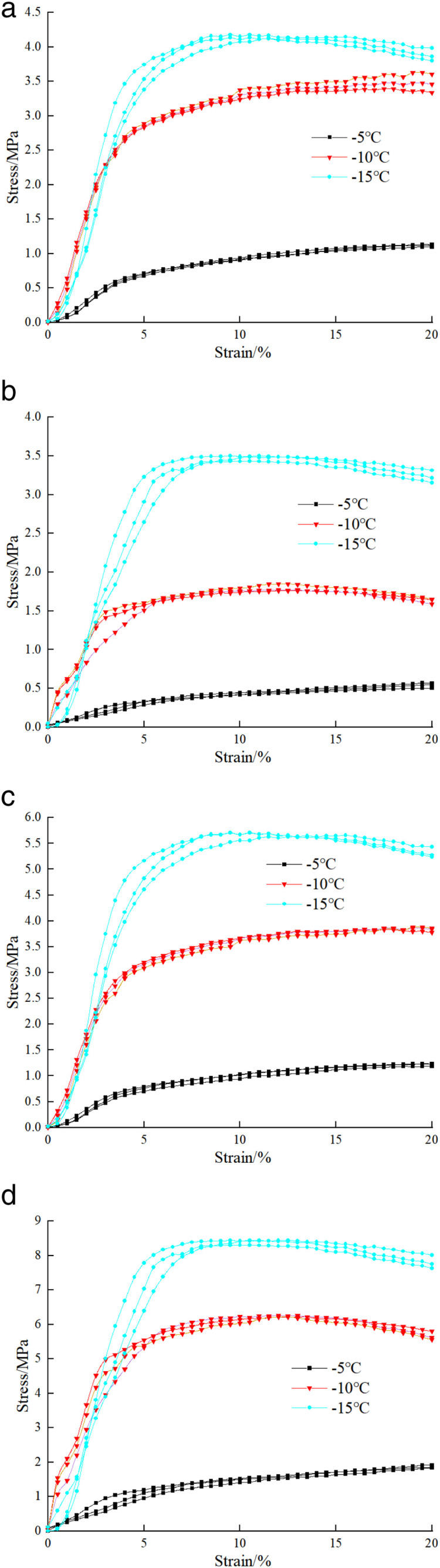
Stress-strain curves. **(a)** Silty clay. **(b)** Mucky soil. **(c)** Residual cohesive soil. **(d)** Fully weathered ignimbrite.

The test results of uniaxial instantaneous compressive strength of frozen soil under different temperature conditions are shown in Table 2, and the relationship curve between uniaxial compressive strength and temperature of frozen soil is shown in Fig 10. It can be seen from the test results that the uniaxial compressive strength of frozen soil has a good positive correlation with temperature, and the strength of frozen soil increases gradually with the decrease of freezing temperature.

**Table 2:** Uniaxial instantaneous compressive strength of frozen soil.

Soil layer	Test temperature					
	−5°C		−10°C		−15°C	
	Uniaxial strength/MPa	Average value/MPa	Uniaxial strength/MPa	Average value/MPa	Uniaxial strength/MPa	Average value/MPa
**silty clay**	1.11	1.11	3.39	3.50	4.15	4.15
	1.09		3.48		4.13	
	1.13		3.63		4.18	
**mucky soil**	0.50	0.54	1.76	1.79	3.50	3.47
	0.55		1.77		3.49	
	0.57		1.85		3.43	
**residual cohesive soil (plastic)**	1.22	1.21	3.82	3.85	5.66	5.69
	1.18		3.86		5.71	
	1.23		3.88		5.70	
**fully weathered ignimbrite (sandy soil-like)**	1.84	1.86	6.21	6.23	8.43	8.39
	1.91		6.26		8.44	
	1.83		6.22		8.30	

The test data were sorted to obtain the relationship curves between axial stress σ and vertical strain ε of each soil sample at −5°C, −10°C and −15°C, as shown in Fig 10.

### 4.4 Uniaxial creep of frozen soil

**Fig 11:**
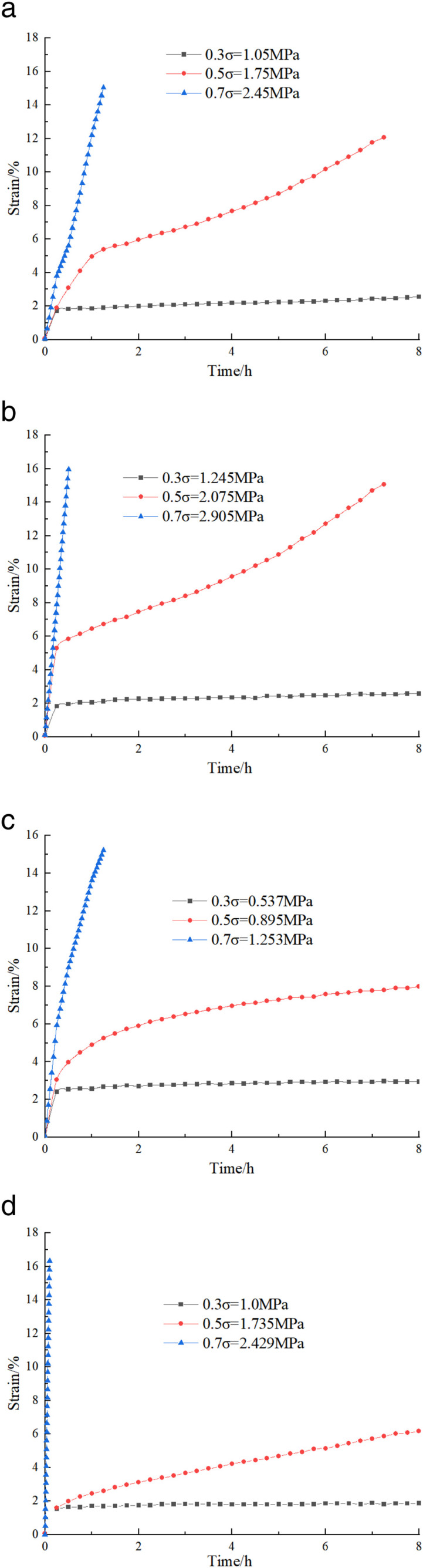
Relationship curves between axial creep strain and time of frozen soil samples under different temperature conditions. **(a)** Uniaxial creep curve of silty clay at −10°C. **(b)** Uniaxial creep curve of silty clay at −15°C. **(c)** Uniaxial creep curve of mucky soil at −10°C. **(d)** Uniaxial creep curve of mucky soil at −15°C.

The relationship curves between axial creep strain and time of silty clay and mucky soil under different temperature conditions are shown in Fig 11.

It can be seen from Fig 11 that the creep curves of frozen soil under different stress levels have different characteristics: the uniaxial creep curve of frozen soil shows two stages under low pressure (0.3σs): instantaneous strain stage and decelerating creep stage; it presents typical three stages under medium pressure (0.5σs): instantaneous strain stage, decelerating creep stage and steady-state creep stage. Under the action of high pressure (0.7σs), most samples directly show accelerated creep in a short time. The analysis of the reasons is as follows: under low pressure (0.3σs), the adjustment of ice crystal structure and pore compaction dominate the deformation, which gradually tends to be stable; under medium pressure (0.5σs), the continuous slip of ice forms steady-state creep; under high pressure (0.7σs), the damage of ice crystal skeleton and soil particle connection is accelerated, leading to the initiation and propagation of microcracks, and the sample quickly enters the accelerated creep stage until failure.

Through horizontal comparison of the creep curves of the four soil samples under the same temperature and stress level, it is found that the creep strain of silty clay is significantly smaller than that of mucky soil, which is related to its lower moisture content, higher initial strength and more stable structure. The temperature effect is significant: the steady-state creep rate at −15°C is much lower than that at −10°C, indicating that lower temperature can effectively inhibit creep deformation by enhancing the cementation of ice and reducing the content of unfrozen water. During on-site construction, for soil layers with high creep sensitivity (such as mucky soil), it is necessary to adopt a lower design temperature or shorter structure exposure time to control the time-dependent deformation and ensure the safety of the existing structure during the under-crossing construction.

### 4.5 Triaxial creep test of frozen soil

**Fig 12:**
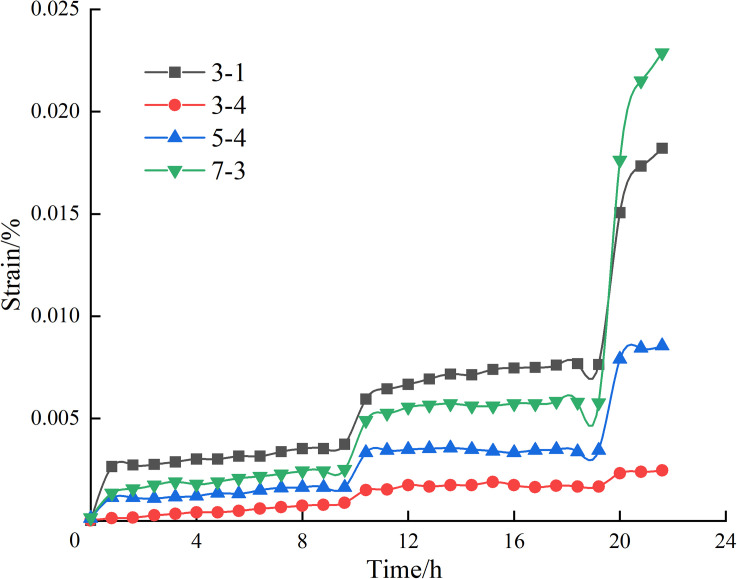
Three-stage creep curves of four types of frozen soil under −10°C and 1MPa confining pressure.

The three-stage creep strain-time curves under 0.3, 0.5 and 0.7 times the peak shear strength at −10°C and 1MPa confining pressure are shown in Fig 12.

It can be seen from Fig 12 that under the shear stress of the first stage (0.3 times the peak shear strength), the maximum strain of 3–1 (silty clay) reaches 0.376%, and the strain of 3–4 (mucky soil) is the smallest; under the shear stress of the second stage (0.5 times the peak shear strength), the maximum strain of 3–1 soil reaches 0.771%, and the strain of 3–4 soil is the smallest; under the shear stress of the third stage (0.7 times the peak shear strength), the maximum strain of 6–2 (fully weathered ignimbrite) reaches 2.285%, and the strain of 3–4 soil is the smallest. The triaxial creep test of frozen soil shows that with the increase of stress level, the creep rate increases gradually, and the creep mode changes from stable creep to accelerated creep. The long-term strength of frozen soil under triaxial stress condition is obtained, which is 0.7 times the triaxial shear strength.

By comparing the results of uniaxial and triaxial creep tests, it is found that the failure time of triaxial samples is significantly delayed under the same stress level, which indicates that the existence of confining pressure restricts the lateral deformation of soil, delays the development of micro damage, and improves the creep life of frozen soil under complex stress state.

### 4.6 Triaxial shear test results of frozen soil

**Fig 13:**
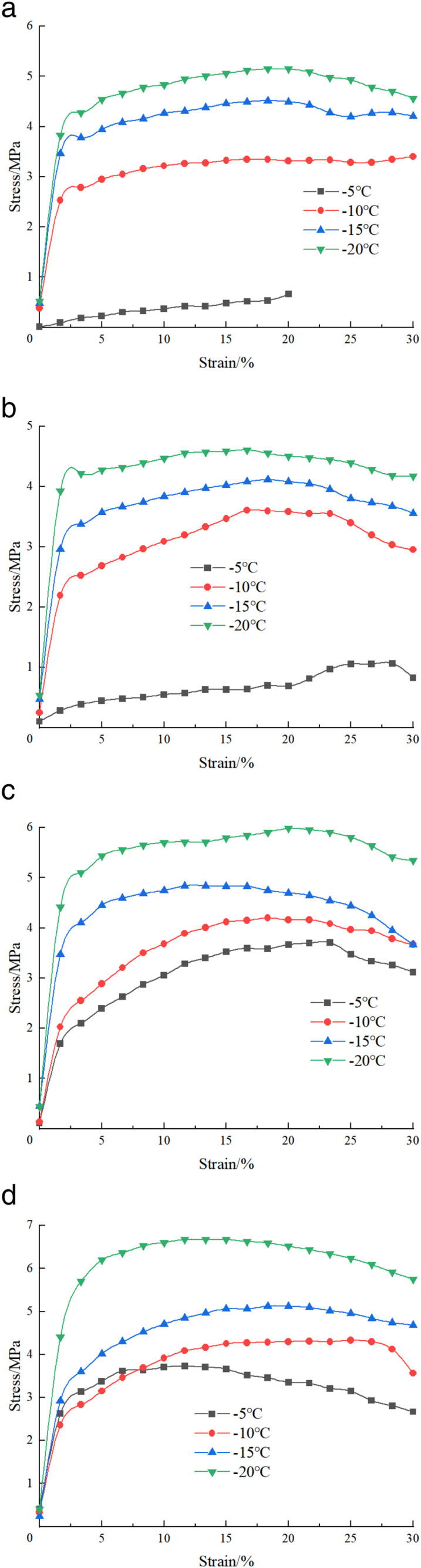
Triaxial shear test results of soil samples at different temperatures under 1MPa confining pressure. **(a)** Silty clay. **(b)** Mucky soil. **(c)** Residual cohesive soil. **(d)** Fully weathered ignimbrite.

**Fig 14:**
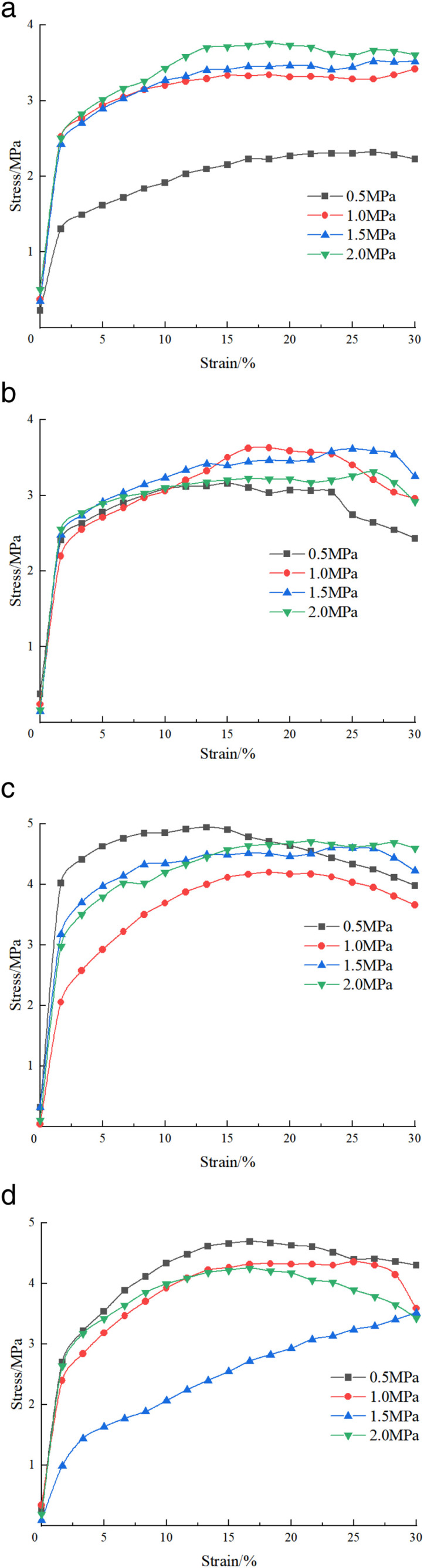
Triaxial shear test results of soil samples with different confining pressures at −10°C. **(a)** Silty clay. **(b)** Mucky soil. **(c)** Residual cohesive soil. **(d)** Fully weathered ignimbrite.

The variation curves of triaxial shear strength of soil samples with freezing temperature under 1MPa confining pressure and with confining pressure at −10°C are shown in Figs 13 and 14 respectively.

It can be seen from Fig 13 that the triaxial shear strength of soil samples under 1MPa confining pressure increases gradually with the decrease of frozen soil temperature. The fundamental reason is that the decrease of temperature leads to the reduction of unfrozen water content and the increase of ice content in the soil, thus enhancing the cementation effect of ice on soil particles and its own shear strength. The strength growth is not linear, and the most significant increase occurs in the range of −5°C to −10°C, which is consistent with the law of sharp decrease of unfrozen water content in this temperature range. The maximum triaxial shear strength of silty clay at −20°C is 5.14MPa. At −5°C, the soil samples are damaged at 20% strain; the maximum triaxial shear strength of mucky soil at −20°C is 4.61MPa; the maximum triaxial shear strength of residual cohesive soil at −20°C is 5.98MPa; the maximum triaxial shear strength of fully weathered ignimbrite (sandy soil-like) at −20°C is 6.67MPa.

Horizontal comparison of the four soil samples shows that the strength ranks as follows: fully weathered ignimbrite > residual cohesive soil > silty clay > mucky soil. The analysis shows that ignimbrite and residual soil have coarser particles and denser structure, providing higher internal friction angle and shear strength; while mucky soil has the lowest strength due to its characteristics of high moisture content and high porosity. In engineering design, different soil layers should be treated differently. For soil layers with low strength (such as mucky soil), lower freezing temperature or additional reinforcement measures should be considered.

It can be seen from Fig 14 that the variation of triaxial shear strength of soil samples with confining pressure at −10°C is slightly different:

(1) The triaxial shear strength of silty clay increases from 2.33MPa to 3.81MPa when the confining pressure increases from 0.5MPa to 2MPa. When the confining pressure increases from 0.5MPa to 1MPa, the triaxial shear strength of silty clay increases greatly, while when it continues to increase to 2MPa, the growth rate of triaxial shear strength of silty clay becomes smaller. This indicates that the confining pressure enhances the friction and interlocking effect between soil particles by limiting the lateral expansion of soil, and the effect is significant in the initial stage. However, with the continuous increase of confining pressure, the marginal effect on strength improvement decreases, which is consistent with the Mohr-Coulomb strength theory.
(2) With the increase of confining pressure, the triaxial shear strength of mucky soil increases first and then decreases. When the confining pressure is 1.5MPa, the maximum strength is 3.24MPa. In general, confining pressure has little effect on the triaxial shear strength of mucky soil at −10°C. The strength decreases after reaching the peak at a certain confining pressure, which may be related to the local damage of soil structure or water redistribution under high pressure. High pore water pressure may weaken the effective stress. In practical engineering, attention should be paid to the potential strength softening risk of this type of soil under high confining pressure environment, and the support design should not rely too much on the strength gain brought by high confining pressure.
(3) The triaxial shear strength of residual cohesive soil shows a trend of first decreasing and then increasing with the increase of confining pressure. When the confining pressure is 2MPa, the maximum strength is 4.34MPa. The reason is that the microcracks in the soil may be activated or the structure is slightly adjusted under medium and low confining pressure, resulting in a temporary decrease in strength. With the continuous increase of confining pressure, the soil is gradually fully compacted, and the strength is restored and improved. For this type of soil, special attention should be paid to the possible strength fluctuation in the middle confining pressure range during freezing construction.
(4) The triaxial shear strength of fully weathered ignimbrite tends to decrease with the increase of confining pressure. When the confining pressure is 1MPa, the triaxial shear strength of fully weathered ignimbrite (sandy soil-like) at −10°C is 4.30MPa. Its strength decreases slightly with the increase of confining pressure, which may be related to the brittle failure of cement between particles or particle breakage under high pressure, indicating that this type of soil does not show typical pressure hardening under high confining pressure, and its strength behavior is closer to brittle materials. In engineering, the support for it should focus on maintaining its integrity rather than simply increasing the confining pressure.

## 5. Conclusions

(1) The thermal conductivity of the four soil samples is different at normal temperature (20°C) and low temperature (−10°C). The fully weathered ignimbrite (sandy soil-like) has the highest thermal conductivity, while the mucky soil has the lowest. The thermal conductivity of soil samples at low temperature is higher than that at normal temperature.
(2) The freezing temperatures of the four soil samples under natural moisture content are as follows: (a) the freezing temperature of silty clay is −1.7°C, (b) the freezing temperature of mucky soil is −1.25°C, (c) the freezing temperature of residual cohesive soil (plastic) is −2.25°C, and (d) the freezing temperature of fully weathered ignimbrite (sandy soil-like) is −0.8°C.
(3) The creep characteristics of frozen soil show significant stress dependence: it mainly presents decelerating creep at low stress level (0.3σs), steady-state creep at medium stress level (0.5σs), and quickly enters the accelerated creep stage until failure at high stress level (0.7σs). Due to the low moisture content and stable structure, the creep deformation of silty clay is much smaller than that of mucky soil. Meanwhile, reducing the temperature (e.g., from −10°C to −15°C) can significantly inhibit the development of creep. Therefore, in practical engineering, it is recommended to adopt lower freezing temperature or strictly control the load exposure time for strata with high creep sensitivity (such as mucky soil), so as to ensure the long-term stability of the frozen wall and the safety of under-crossing construction.
(4) The triaxial shear test results of frozen soil show that the triaxial shear strength of frozen soil is significantly affected by temperature and confining pressure, and has obvious soil dependence. With the decrease of temperature, the strength of all types of soil increases, especially in the range of −5°C to −10°C. The strength of the four soils ranks as follows: fully weathered ignimbrite > residual cohesive soil > silty clay > mucky soil. The influence of confining pressure on strength varies with soil types: silty clay shows typical pressure hardening, but the increase amplitude weakens with the increase of confining pressure; mucky soil and ignimbrite show atypical responses, with the strength decreasing after a certain confining pressure or continuously decreasing, which is related to structural damage, pore water pressure or brittle fracture of cement. Therefore, in artificial ground freezing engineering, differentiated control should be implemented according to the characteristics of soil layers: lower freezing temperature can be adopted for strength-sensitive strata, while for strata with abnormal confining pressure response, the support pressure should be strictly controlled and monitoring should be strengthened, so as to achieve the optimal balance between safety and economy.
